# The diagnostic value of ^18^F–FDG-PET/CT and MRI in suspected vertebral osteomyelitis – a prospective study

**DOI:** 10.1007/s00259-017-3912-0

**Published:** 2017-12-19

**Authors:** Ilse J. E. Kouijzer, Henk Scheper, Jacky W. J. de Rooy, Johan L. Bloem, Marcel J. R. Janssen, Leon van den Hoven, Allard J. F. Hosman, Leo G. Visser, Wim J. G. Oyen, Chantal P. Bleeker-Rovers, Lioe-Fee de Geus-Oei

**Affiliations:** 1 Department of Internal Medicine and Infectious Diseases, Radboud University Medical Center, P.O. Box 9101, 6500 HB Nijmegen, the Netherlands; 2 MIRA Institute for Biomedical Technology and Technical Medicine, Biomedical Photonic Imaging Group, University of Twente, Enschede, the Netherlands; 3 Department of Internal Medicine and Infectious Diseases, Leiden University Medical Center, Leiden, the Netherlands; 4 Department of Radiology and Nuclear Medicine, Radboud University Medical Center, Nijmegen, the Netherlands; 5 Department of Radiology, Leiden University Medical Center, Leiden, the Netherlands; 6 Technical Medicine, University of Twente, Enschede, the Netherlands; 7 Department of Orthopedic Surgery, Radboud University Medical Center, Nijmegen, the Netherlands; 8 Department of Nuclear Medicine, The Institute of Cancer Research and Royal Marsden NHS Foundation Trust, London, UK

**Keywords:** Vertebral osteomyelitis, ^18^F–Fdg-pet/ct, MRI, Abscesses

## Abstract

**Purpose:**

The aim of this study was to determine the diagnostic value of ^18^F–fluorodeoxyglucose (FDG) positron emission tomography and computed tomography (PET/CT) and magnetic resonance imaging (MRI) in diagnosing vertebral osteomyelitis.

**Methods:**

From November 2015 until December 2016, 32 patients with suspected vertebral osteomyelitis were prospectively included. All patients underwent both ^18^F–FDG-PET/CT and MRI within 48 h. All images were independently reevaluated by two radiologists and two nuclear medicine physicians who were blinded to each others’ image interpretation. ^18^F–FDG-PET/CT and MRI were compared to the clinical diagnosis according to international guidelines.

**Results:**

For ^18^F–FDG-PET/CT, sensitivity, specificity, PPV, and NPV in diagnosing vertebral osteomyelitis were 100%, 83.3%, 90.9%, and 100%, respectively. For MRI, sensitivity, specificity, PPV, and NPV were 100%, 91.7%, 95.2%, and 100%, respectively. MRI detected more epidural/spinal abscesses. An important advantage of ^18^F–FDG-PET/CT is the detection of metastatic infection (16 patients, 50.0%).

**Conclusion:**

^18^F–FDG-PET/CT and MRI are both necessary techniques in diagnosing vertebral osteomyelitis. An important advantage of ^18^F–FDG-PET/CT is the visualization of metastatic infection, especially in patients with bacteremia. MRI is more sensitive in detection of small epidural abscesses.

## Introduction

Vertebral osteomyelitis is a severe infection of the spine and its prevalence is increasing in our aging society [1]. Common complications of vertebral osteomyelitis are epidural, spinal, or psoas abscesses. Epidural/spinal abscesses may result in paraplegia if they are not diagnosed and treated promptly. One third of patients with vertebral osteomyelitis suffer from residual spinal dysfunction or persistent pain after recovery [2, 3]. Therefore, early and accurate detection of vertebral osteomyelitis is necessary for improved outcome [4]. Symptoms and signs of vertebral osteomyelitis, however, are often unspecific and diagnosis is difficult. Magnetic resonance imaging (MRI) is most often used as imaging technique in diagnosing vertebral osteomyelitis [5] with a reported sensitivity and specificity of more than 90% [6, 7]. Disadvantages of MRI are artifacts due to metallic implants, occasional similarities between vertebral osteomyelitis and degenerative disease [8], and reduced sensitivity in patients with short duration of symptoms [8, 9].

Combined ^18^F–fluorodeoxyglucose (FDG) positron emission tomography and computed tomography (PET/CT) is increasingly used in diagnosing infectious diseases. In patients suspected of vertebral osteomyelitis, the first studies on the value of ^18^F–FDG-PET (without combined CT) showed high sensitivity and specificity up to 100% [10–12]. The value of ^18^F–FDG-PET/CT in patients with vertebral osteomyelitis has been studied [13] and also compared to MRI [14, 15]. However, in these studies, the time between start of symptoms and moment of imaging was not mentioned and time between ^18^F–FDG-PET/CT and MRI is also unknown. The purpose of this study was to prospectively compare the diagnostic value of MRI and ^18^F–FDG-PET/CT in diagnosing vertebral osteomyelitis and its complications with a maximum time interval of 48 h between imaging techniques.

## Materials and methods

### Patients

In this prospective study at the Radboud University Medical center and at the Leiden University Medical Center, all adult patients with clinically suspected vertebral osteomyelitis from November 2015 to December 2016 were included. Vertebral osteomyelitis was suspected in case of fever and back pain, in case of bacteremia and back pain, or when there was an increased C-reactive protein (CRP) or erythrocyte sedimentation rate (ESR) and back pain. Exclusion criteria were pregnancy, known metastases in the spine, poorly regulated diabetes mellitus, too ill for transportation to ^18^F–FDG-PET/CT and/or MRI, and absolute contra-indications for MRI. Both ^18^F–FDG-PET/CT and MRI were performed in all patients within 48 h from each other and with a preferable interval of no more than 24 h. In case of negative blood cultures, CT-guided biopsy of the spine was strongly recommended by the study physician in all patients. If the CT-guided biopsy was inconclusive, open biopsy by the orthopedic surgeon was strongly recommended. MRI was repeated after 2 weeks if ^18^F–FDG-PET/CT showed abnormalities suggestive of vertebral osteomyelitis, and the first MRI was negative. The institutional review board approved this study and informed consent was waived.

### ^18^F–FDG-PET/CT and MRI

Two integrated PET/CT scanners (Biograph 40 mCT; Siemens Healthcare and Gemini TF64; Philips) were used. All patients were on a low carbohydrate-fat allowed diet 24 h before ^18^F–FDG-PET/CT was performed, and they fasted 6 h before ^18^F–FDG-injection. Blood glucose levels were required to be less than 12 mmol/l in all patients, including in diabetic patients. One hour after intravenous injection of a 3.3 MBq/kg average dose of ^18^F–FDG (Mallinckrodt Pharmaceuticals, Petten, the Netherlands or IBA Molecular, Amsterdam, the Netherlands), whole-body low-dose CT scan was acquired for anatomic correlation and attenuation correction of the PET data. MRI of the spine was performed using 1.5 T systems (Siemens, Erlangen, Germany, and Philips, Best, the Netherlands).

### Follow-up

Patients who were diagnosed with vertebral osteomyelitis based on at least one imaging study and/or blood or tissue culture results were treated with antibiotics for 6 weeks according to the IDSA (Infectious Diseases Society of America) guideline for vertebral osteomyelitis [16]. In case of other infectious foci with subsequent indication for longer duration of antibiotic treatment (i.e., vascular graft infection or prosthetic joint infection), patients were treated longer than 6 weeks.

Three months after inclusion, all surviving patients visited the outpatient clinic for evaluation of symptoms. Patients were considered to be cured when there were no symptoms or signs of infection (i.e., fever, persistently increased CRP, persistent positive blood cultures, persistent back pain) after discontinuation of antibiotic treatment. Persistent infection was considered to be present when patients were still treated for vertebral osteomyelitis at three month follow-up without resolution of the described symptoms. Relapse of infection was defined as a second episode of vertebral osteomyelitis with the same causative micro-organism after completion of adequate antibiotic treatment of at least six weeks duration. Mortality was considered to be infection related when a patient died during the episode of vertebral osteomyelitis with persistent signs or symptoms of systemic infection or after relapse without another possible cause of death.

### Evaluation of imaging

All ^18^F–FDG-PET/CT scans were evaluated after the inclusion period by two independent nuclear medicine physicians without knowledge of the clinical context of patients by using the score as mentioned in Table 1. Disagreements were resolved by consensus afterwards. All MRI scans were evaluated by two independent radiologists without knowledge of the clinical context of patients. Both radiologists had many years of experience and were specifically trained for musculoskeletal imaging. For evaluation of MRI the score as mentioned in Table 1 was used. Disagreements were resolved by consensus afterwards. The original reports and the revised reports were evaluated using the following parameters: sensitivity, specificity, positive predictive value (PPV), and negative predictive value (NPV). The results of imaging (both original and revised reports) were compared with the clinical diagnosis as reference standard according to the IDSA guideline for vertebral osteomyelitis [16]. As the gold standard for the diagnosis, we defined vertebral osteomyelitis, in accordance with the IDSA guideline, as new back pain plus positive tissue/blood culture plus at least one positive imaging outcome. Original imaging reports were compared to revised imaging reports to investigate the need of an expert opinion in evaluation of imaging in suspected vertebral osteomyelitis and also in relation to the duration of symptoms. The primary outcome parameters were sensitivity, specificity, PPV, and NPV of both original and revised reports of ^18^F–FDG-PET/CT and MRI for the diagnosis of vertebral osteomyelitis. A secondary outcome parameter was detection of epidural/spinal abscesses, paravertebral abscesses, and psoas abscesses.

**Table 1:** Five-point grading score for assessment of ^18^F–FDG-PET/CT and MRI in suspected vertebral osteomyelitis

	^18^F–FDG-PET/CT	MRI
Score 0^a^	Normal findings and physiological ^18^F–FDG distribution	Normal findings except for degeneration
Score 1^a^	Minimal increased ^18^F–FDG uptake compared to normal bone marrow ^18^F–FDG uptake	Minimal decreased SI T1 and increased SI T2 and enhancement in intervertebral or paravertebral region compared to normal bone marrow
Score 2A^b^	Increased ^18^F–FDG uptake with a linear or disciform pattern in intervertebral disc space	Decreased SI T1 and increased SI T2 and enhancement with linear or disciform pattern at intervertebral disc space
Score 2B^b^	Increased ^18^F–FDG uptake in only ossal structures without pathological changes in intervertebral discs	Decreased SI T1 and increased SI T2 and enhancement in only ossal structures, without changes in intervertebral discs
Score 3^c^	Increased ^18^F–FDG uptake with a linear or disciform pattern in intervertebral disc space and involvement of endplate (or adjacent vertebrae)	Decreased SI T1 and increased SI T2 at intervertebral disc space and involvement of endplate (or adjacent vertebrae)
Score 4^c^	Increased ^18^F–FDG uptake with a linear or disciform pattern in intervertebral disc space and involvement of endplate with surrounding soft tissue abscesses	Decreased SI T1 and increased SI T2 at intervertebral disc space and involvement of endplate with surrounding soft tissue abscesses.

^a^Score 0 and 1 were considered as normal or aspecific and excluded vertebral osteomyelitis

^b^Score 2A was considered as discitis and score 2B as osteomyelitis (without discitis)

^c^Score 3 and 4 were considered as vertebral osteomyelitis

### Statistics

All data were collected in a structured database using SPSS statistics (version 20.0; IMB Corp.) and diagnostic value of both original and revised reports of ^18^F–FDG-PET/CT and MRI was determined by calculating sensitivity, specificity, PPV, NPV, and 95% confidence interval (CI).

## Results

### Patients

A total of 32 patients were included. Baseline characteristics of all patients are shown in Table 2. All patients included in the study had community-acquired infections. Vertebral osteomyelitis was diagnosed in 20 patients and this diagnosis was made according to the IDSA guideline [16]. In 12 patients without vertebral osteomyelitis the following diagnoses were made based on imaging and clinical findings; degenerative spinal disease in five patients, and single patients had tendomyalgia, spinal metastases of urothelial carcinoma, infected aortic aneurysm, pyelonephritis, Charcot spine, infected spinal osteosynthesis of sacroiliac joints without vertebral osteomyelitis, and immobilization due to severe dyskeratosis follicularis (Darier’s disease). Four patients with vertebral osteomyelitis died of whom three were infection-related, because these patients died due to complications of the infection, one patient died due to severe myelodysplastic syndrome. No relapses occurred within 3 months after treatment. Treatment was continued after 3 months in eight patients (38.1%). Two patients without the diagnosis of vertebral osteomyelitis died, one patient due to metastasized urothelial carcinoma and one patient due to liver cirrhosis.

**Table 2:** Baseline characteristics of patients with and without vertebral osteomyelitis

	All patients (*n* = 32)	Patients with vertebral osteomyelitis (*n* = 20)	Patients without vertebral osteomyelitis (*n* = 12)
Male (%)	22 (68.8)	16 (80.0)	6 (50.0)
Age (range)	66.8 (43–92)	70.2 (43–92)	61.1 (46–81)
Medical history			
- Malignancy (%)	4 (12.5)	2 (10.0)	2 (16.7)
- Immunocompromized (%)	2 (6.3)	2 (10.0)	0
- Diabetes mellitus (%)	4 (12.5)	3 (15.0)	1 (8.3)
- Spinal implants (%)	2 (6.3)	0	2 (16.7)
- Spinal surgery <1y (%)	1 (3.1)	0	1 (8.3)
- Other implants^a^ (%)	10 (31.3)	6 (30.0)	4 (33.3)
Fever (%)	23 (71.9)	14 (70.0)	8 (66.7)
Increased CRP/ESR (%)	31 (96.9)	20 (100)	11 (91.7)
Positive blood culture^b^ (%)	26 (81.3)	18 (85.7)	8 (72.7)

^a^Other implants: vascular graft (*n* = 4), heart valve prosthesis (n = 2), total hip prosthesis (*n* = 2), double J stent (*n* = 1), sacral neuromodulator (*n* = 1)

^b^11 patients (42.3%) had *Staphylococcus aureus* bacteremia, one patient (3.8%) had bacteremia with coagulase-negative *Staphylococcus*, eight patients (30.8%) had bacteremia with *Streptococcus species*, two patients (7.7%) with *Enterococcus faecalis*, two patients (7.7%) with *Escherichia coli*, and one patient (3.8%) with *Klebsiella pneumoniae,* and one patient (3.8%) with *Aerococcus urinae*

### MRI and ^18^F–FDG-PET/CT

In eight patients (25.0%) MRI and ^18^F–FDG-PET/CT were performed on the same day, in 18 patients (56.3%) within 24 h, and in six patients (18.8%) after 24 h but within 48 h. The median time interval between first symptoms and imaging was 24.9 days with a range of 3–120 days. Fifteen patients (46.9%) had symptoms less than 14 days and six patients (18.8%) less than seven days.

Of all original reports of MRI scans, 29 reports had the same conclusion compared to reevaluation by an expert panel (90.6%). Of all original reports of ^18^F–FDG-PET/CT, 31 reports had the same conclusion compared to reevaluation by an expert panel (96.9%). In one patient, the original ^18^F–FDG-PET/CT report was true negative, while reevaluation was false positive for vertebral osteomyelitis. MRI did not show vertebral osteomyelitis in this patient. This patient had chronic Q fever with an infected endovascular aortic repair (EVAR) without vertebral osteomyelitis.

**Fig. 1:**
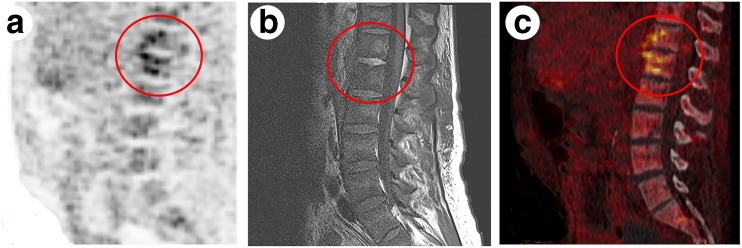
Imaging of the lumbar spine of a 43-year-old woman on haemodialysis who was admitted because of *S. aureus* bacteremia and back pain. ^18^F–FDG-PET (**a**) and ^18^F–FDG-PET/CT (**c**) showing increased ^18^F–FDG uptake of T12-L1 (score 4). T1-weighted Gd-chelate enhanced MRI (**b**) showing subtle enhancement of the intervertebral disc at level T12-L1 and of the perivertebral soft tissues and subtle interruption of the anterior endplates of T12 and L1 (score 4). The original MRI report was false-negative indicating a psoas abscess but no vertebral osteomyelitis. Original ^18^F–FDG-PET/CT and also reevaluated ^18^F–FDG-PET/CT and MRI did show vertebral osteomyelitis on level T12-L1. Repeated MRI after 2 weeks also confirmed the diagnosis of vertebral osteomyelitis

**Fig. 2:**
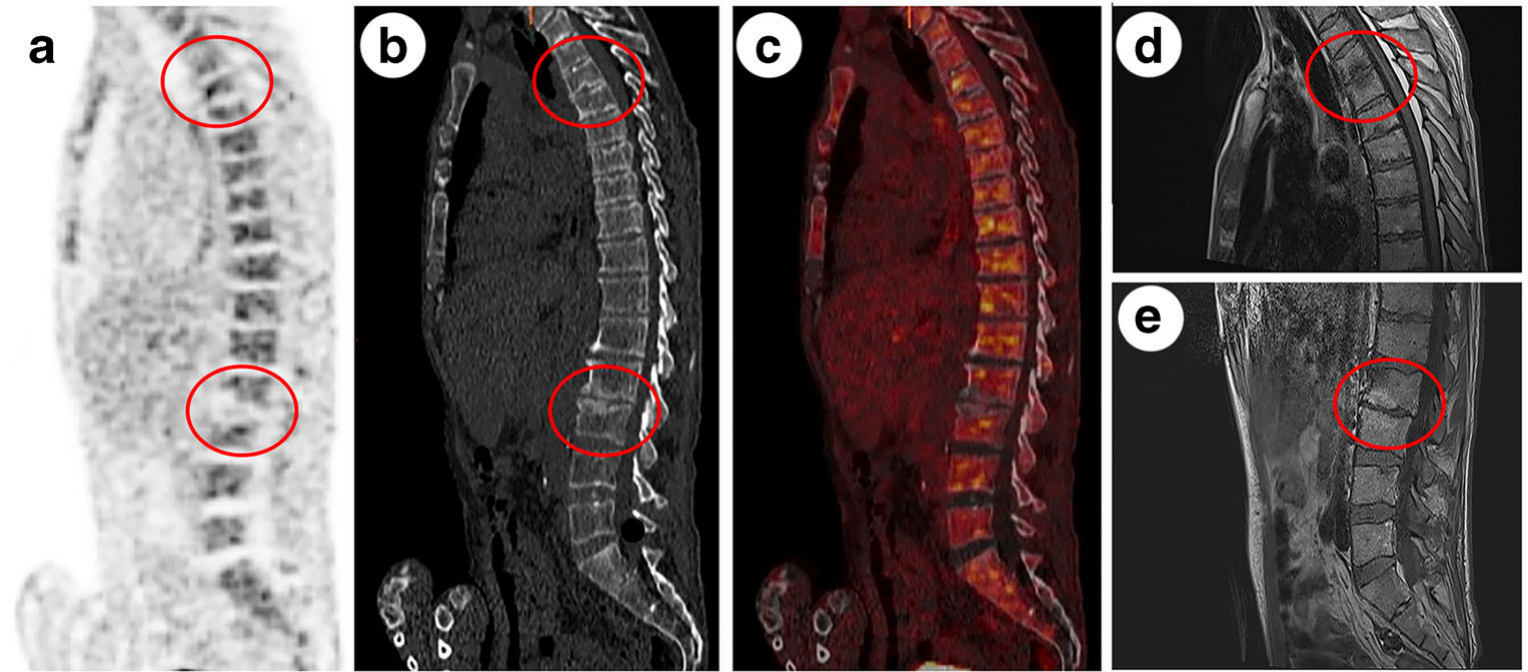
^18^F–FDG-PET/CT and MRI of a 61-year-old man who was admitted with *S. aureus* endocarditis and back pain. ^18^F–FDG-PET (**a**), CT (**b**), and ^18^F–FDG-PET/CT (**c**) showing degenerative changes on level T3-T4 and L2-L3. T1-weighted Gd-chelate enhanced MRI (**d**, **e**), pre- and postcontrast, showing Modic type 1 degenerative changes at level T3–4 and L2-L3. In the original MRI report a diagnosis of vertebral osteomyelitis on the levels T3-T4 and L2-L3 was made, ^18^F–FDG-PET/CT was negative for vertebral osteomyelitis. Reevaluation of ^18^F–FDG-PET/CT and MRI were both negative (score 1) and long-term follow-up confirmed that the back pain could be attributed to degenerative changes of the spine

Two original MRI reports discarded a diagnosis of vertebral osteomyelitis while reevaluation did show vertebral osteomyelitis. These two patients had back pain for 10 and 7 days before the first moment of imaging and repeated MRI after 14 days confirmed vertebral osteomyelitis in both (Fig. 1). In one patient with *S. aureus* bacteremia and endocarditis, the original MRI report concluded vertebral osteomyelitis and reevaluation excluded vertebral osteomyelitis. In this patient, back pain was asserted as degenerative disc disease as stated by the reevaluated MRI report and ^18^F–FDG-PET/CT reports (Fig. 2). When comparing diagnostic values of imaging performed within 14 days after start of symptoms and imaging performed after 14 days after start of symptoms, diagnostic values for MRI performed within 14 days were higher for revised imaging reports compared to original MRI reports (Table 3). For ^18^F–FDG-PET/CT and all imaging performed after 14 days after start of symptoms there were no important differences between original and revised results.

**Table 3:** Diagnostic results of original and revised reports of imaging in 20 patients with early (< 14 days of symptoms) and late (> 14 days of symptoms) stage of vertebral osteomyelitis

	MRI		^18^F–FDG-PET/CT	
	Original report	Revised report	Original report	Revised report
< 14 days				
Sensitivity	77.8% (40.0–97.2)	100% (66.4–100)	100% (66.4–100)	100% (66.3–100)
Specificity	83.3% (35.9–99.6)	100% (54.1–100)	100% (54.1–100)	83.3% (35.9–99.6)
PPV	87.5% (53.1–97.7)	100% (70.1–100)	100% (70.1–100)	90.0% (60.1–98.2)
NPV	71.4% (41.6–89.9)	100% (61.0–100)	100% (61.0–100)	100% (56.6–100)
> 14 days				
Sensitivity	100% (71.5–100)	100% (71.5–100)	100% (71.5–100)	100% (71.5–100)
Specificity	83.3% (35.9–99.6)	83.3% (35.9–99.6)	83.3% (35.9–99.6)	83.3% (35.9–99.6)
PPV	91.7% (64.8–98.5)	91.7% (64.8–98.5)	91.7% (64.8–98.5)	91.7% (64.8–98.5)
NPV	100% (56.6–100)	100% (56.6–100)	100% (56.6–100)	100% (56.6–100)

For MRI and ^18^F–FDG-PET/CT in diagnosing vertebral osteomyelitis, overall sensitivity, specificity, PPV, and NPV are shown in Table 4. In one patient without the clinical diagnosis of vertebral osteomyelitis but with a Charcot spine ^18^F–FDG-PET/CT and MRI which were both false-positive, showing signs of vertebral osteomyelitis with surrounding abscesses. Biopsy of the soft tissue involvement was performed and culture was negative as well as PCR (polymerase chain reaction) for *Coxiella burnetii* and bacterial 16S rDNA PCR. This patient is in good condition without any use of antibiotics, now eight months after imaging.

**Table 4:** Diagnostic results of ^18^F–FDG-PET/CT and MRI for diagnosing vertebral osteomyelitis

	^18^F–FDG-PET/CT (n = 32)	MRI (*n* = 32)
Diagnosis of vertebral osteomyelitis	20	20
No. of true-positive results	20	20
No. of false-positive results	2	1
No diagnosis of vertebral osteomyelitis	12	12
No. of true-negative results	10	11
No. of false-negative results	0	0
Sensitivity (95% CI)	100% (79.9–100)	100% (79.9–100)
Specificity (95% CI)	83.3% (50.9–97.1)	91.7% (60.0–99.6)
PPV (95% CI)	90.9% (69.4–98.4)	95.2% (74.1–99.8)
NPV (95% CI)	100% (65.5–100)	100% (67.9–100)

### Diagnosing abscesses

Vertebral osteomyelitis with abscesses was found in 11 out of 20 patients. Five patients had epidural and spinal abscesses, nine patients had paravertebral abscesses, and four patients had psoas abscesses. With MRI all five and with ^18^F–FDG-PET/CT only one epidural/spinal abscesses were detected. With ^18^F–FDG-PET/CT five out of nine paravertebral abscesses and with MRI seven out of nine abscesses were detected. ^18^F–FDG-PET/CT detected all four psoas abscesses. MRI detected three out of four psoas abscesses, as on one MRI the iliopsoas was not fully included in the field of view.^18^F–FDG-PET/CT detected metastatic infection in 16 patients (50.0%) and ^18^F–FDG-PET/CT was the first to localize infectious foci other than vertebral osteomyelitis in 14 of these patients (87.5%). Localizations of metastatic infection were joints (43.8%), pulmonary foci (18.8%), soft tissue (18.8%), endovascular (12.5%), and spleen (6.3%).

## Discussion

MRI and ^18^F–FDG-PET/CT are both valuable in diagnosing vertebral osteomyelitis. Our study shows high sensitivity and specificity for both imaging techniques, without a significant difference between ^18^F–FDG-PET/CT and MRI.

Earlier studies on the diagnosis of vertebral osteomyelitis reported a sensitivity and specificity of 83% and 88% for ^18^F–FDG-PET/CT and 94% and 38% for MRI [14] and a sensitivity and specificity of 82% and 100% for ^18^F–FDG-PET/CT and 75% and 72% for MRI [15]. Smids et al. [17] retrospectively investigated the diagnostic value of ^18^F–FDG-PET/CT and MRI in patients suspected of vertebral osteomyelitis and reported a sensitivity and specificity of 96% and 95% for ^18^F–FDG-PET/CT and 67% and 84% for MRI. This study also showed that the diagnostic accuracy for MRI improved when MRI was performed at least 14 days after start of symptoms compared to MRI performed within 14 days after onset of symptoms (82% and 58%, respectively). In the study of Smids et al., there was no significant difference in accuracy in relation to the moment of imaging for ^18^F–FDG-PET/CT (94% and 97%, respectively) [17]. In the current study, original reports of MRI performed within 14 days showed, although not significantly, lower diagnostic value compared to when MRI was performed after 14 days after start of symptoms (Table 3). However, revision of MRI by an expert panel reversed these differences. This emphasizes the importance of an expert panel for assessment of MRI in suspected vertebral osteomyelitis. Overall, we found a higher diagnostic value for MRI in suspected vertebral osteomyelitis in the current study compared to MRI studies published earlier [6, 7]. This might be partly due to the fact that reevaluation was performed by a panel of experts on musculoskeletal imaging including vertebral osteomyelitis and by using a structured scoring system (Table 1). Because in the study of Smids et al., only original reports of imaging were used (reflecting daily clinical practice), the conclusion of MRI having a lack of accuracy in the very early stage of vertebral osteomyelitis might change when revision of all imaging would have been performed by an expert panel using a structured scoring system. This is an important message for clinical practice, as in case of a highly suspected vertebral osteomyelitis with negative MRI, an expert opinion is highly recommended, especially in case of a short duration of symptoms.

In our study, MRI and ^18^F–FDG-PET/CT were false-positive in a patient with a Charcot spine. Charcot spine, or neuropathic arthropathy, is a known condition to be mistaken for infection on MRI [18].

In the study of Smids et al. [17], MRI was the modality of choice to diagnose epidural and spinal abscesses with a sensitivity of 93%. ^18^F–FDG-PET/CT showed higher sensitivity in diagnosing paravertebral (94%) and psoas abscesses (100%) compared to MRI (61% and 63%, respectively). Our study confirmed that MRI is more valuable in detecting epidural and spinal abscesses compared to ^18^F–FDG-PET/CT.

An important advantage of ^18^F–FDG-PET/CT compared to MRI is that metallic implants are no contraindication and do not cause severe artifacts. Furthermore, ^18^F–FDG-PET/CT imaging detected metastatic infection that often needed further interventions and treatment. ^18^F–FDG-PET/CT has proven its effectiveness in patients with Gram-positive bacteremia and infective endocarditis for detecting metastatic infection with a reduction of relapse and mortality rates [19, 20]. In our study, ^18^F–FDG-PET/CT detected metastatic foci in 50.0% of patients, 87.5% of those foci being asymptomatic. Because we did not use whole-body MRI, MRI was not able to detect metastatic infection in this study. ^18^F–FDG-PET/CT could also differentiate between infection and degeneration [11]. Degeneration may occasionally resemble infectious vertebral osteomyelitis on MRI because of the presence of bone marrow edema, which may make MRI interpretation challenging [18]. Assessment of ^18^F–FDG-PET/CT is, due to the clear guidance of increased ^18^F–FDG uptake, more straight forward than assessment of MRI, which is an important advantage in daily clinical practice.

The new imaging technique ^18^F–FDG-PET combined with MRI could be an excellent combination of ^18^F–FDG-PET/CT and MRI and thereby combining the high diagnostic value, detection of metastatic infection and small abscesses. The first study on the value of ^18^F–FDG-PET/MRI in patients suspected of vertebral osteomyelitis was published by Fahnert et al. [21]. Sensitivity, specificity, PPV, and NPV for ^18^F–FDG-PET/MRI in the study of Fahnert et al. were 100%, 88%, 86%, and 100%, respectively, and they concluded ^18^F–FDG-PET/MRI increases the diagnostic certainty for the detection of vertebral osteomyelitis. Diagnostic value for detection of abscesses was not reported. In the study of Fahnert et al., only patients with earlier inconclusive MRI were included.

In our study, assessment of ^18^F–FDG-PET/CT and MRI was performed using a 5-point grading score (Table 1). In all other studies performed on the value of ^18^F–FDG-PET/CT in suspected vertebral osteomyelitis, no structured grading score was used. Our 5-point grading score could be a practical approach for assessment of both ^18^F–FDG-PET/CT and MRI in suspected vertebral osteomyelitis to provide a more structured evaluation of imaging.

In conclusion, ^18^F–FDG-PET/CT and MRI are both necessary techniques in diagnosing vertebral osteomyelitis. An important advantage of ^18^F–FDG-PET/CT is insensitivity to metal artifacts, the large field of view allowing diagnosis of regional abscesses (that can be missed on small field of view MRI) and metastatic infections, especially in patients with bacteremia. MRI is more sensitive in detection of small epidural abscesses. Integrated ^18^F–FDG-PET/MRI in a ‘one-stop-shop’ combines these qualities and could, therefore, become the imaging technique of choice in suspected vertebral osteomyelitis.
